# Lipidomic Analysis Reveals Branched-Chain and Cyclic Fatty Acids from *Angomonas deanei* Grown under Different Nutritional and Physiological Conditions

**DOI:** 10.3390/molecules29143352

**Published:** 2024-07-17

**Authors:** Arquimedes Paixão Santana-Filho, Aramís José Pereira, Letícia Adejani Laibida, Normanda Souza-Melo, Wanderson Duarte DaRocha, Guilherme Lanzi Sassaki

**Affiliations:** Departamento de Bioquímica e Biologia Molecular, Universidade Federal do Paraná, Curitiba 81531-980, PR, Brazil; santana@ufpr.br (A.P.S.-F.); aramis.jpereira@yahoo.com.br (A.J.P.);

**Keywords:** branched fatty acids, cyclic fatty acids, gas chromatography–mass spectrometry

## Abstract

*Angomonas deanei* belongs to *Trypanosomatidae* family, a family of parasites that only infect insects. It hosts a bacterial endosymbiont in a mutualistic relationship, constituting an excellent model for studying organelle origin and cellular evolution. A lipidomic approach, which allows for a comprehensive analysis of all lipids in a biological system (lipidome), is a useful tool for identifying and measuring different expression patterns of lipid classes. The present study applied GC-MS and NMR techniques, coupled with principal component analysis (PCA), in order to perform a comparative lipidomic study of wild and aposymbiotic *A. deanei* grown in the presence or absence of FBS. Unusual contents of branched-chain iso C17:0 and C19:0-*cis*-9,10 and-11,12 fatty acids were identified in *A. deanei* cultures, and it was interesting to note that their content slightly decreased at the log phase culture, indicating that in the latter growth stages the cell must promote the remodeling of lipid synthesis in order to maintain the fluidity of the membrane. The combination of analytical techniques used in this work allowed for the detection and characterization of lipids and relevant contributors in a variety of *A. deanei* growth conditions.

## 1. Introduction

When it comes to protozoan–host interactions, the cell membrane, which constitutes the main interface between cells, provides not only a site for contact and invasion but also a mechanism for evading host defense mechanisms. Particularly in the case of symbiotic associations, the membrane environment plays a specific role in the exchange of metabolites and nutrients. *Angomonas deanei* (previously known as *Crithidia deanei*) [1,2] is a trypanosomatid that infects only insects. It may be grown in vitro with low nutritional requirements and hosts a β-proteobacterial endosymbiont [3]. The endosymbiont maintains a mutualistic relationship with the host protozoan [4], thus constituting an excellent model for studying organelle origin and cellular evolution [2,5,6,7]. Recently, Motta et al. [8] published the *A. deanei* genome and analyzed the predicted protein sequences of *A. deanei* and its symbiont, clarifying evolutionary aspects and providing information that led to a better understanding of this symbiotic relationship. In this symbiotic relationship, intense metabolic exchanges occur between both partners, the bacterium providing essential enzymes that complete important biosynthetic pathways of the protozoa (such as heme and ornithine synthesis) and in exchange receiving from the host suitable physical conditions and energy supply [1,9,10,11,12]. The symbiont is enclosed by two membranes and exhibits a reduced cell wall, essential for bacterial division and morphological maintenance [13]. Sterols are absent in symbiont membranes whose major phospholipid is cardiolipin, followed by similar amounts of phosphatidylcholine, phosphatidylethanolamine and a lesser proportion of phosphatidylinositol [14]. The endosymbiont enhances protozoan phospholipid production and depends in part on its host cell to obtain phosphatidylcholine [15]. A study conducted by Freitas-Junior et al. [16] showed the effects of a phosphatidylcholine biosynthesis inhibitor on mitochondrion and symbiotic fractions of *A. deanei*, reinforcing the idea that an intense metabolic exchange occurs between the host trypanosomatid and structures of symbiotic origin.

To date, few studies have focused on evaluating the lipid composition of *A. deanei* under various nutritional conditions; moreover, little information is available regarding how the lipid composition of this trypanosomatid changes when it is grown in the absence of this endosymbiont. Since *A. deanei* is not able to synthesize cholesterol, analyzing the lipid changes when grown in the absence of FBS would also provide significant insights regarding lipid metabolism. As a non-destructive technique, Nuclear Magnetic Resonance (NMR) can be used in the preliminary characterization of lipid metabolites, while multivariate analysis methodologies such as principal component analysis (PCA) can be used subsequently to point out the main contributors that distinguish cell lines or experimental data sets [17]. Furthermore, the current available data applying modern analytical techniques to lipid composition of *Trypanosomatidae* representants are insufficient, resulting in a need for researchers in this area to provide up-to-date data that can be used as a reference for comparative studies. The growing field encompassing neural networks and machine learning could greatly benefit from these analytical data to perform automated quantitative and qualitative lipidomic analysis with NMR raw data from different matrices. [18,19]. This study applied GC-MS and NMR techniques, coupled with principal component analysis (PCA), in order to characterize the lipidomic profile of *A. deanei* grown in the presence and absence of fetal bovine serum, as well as in aposymbiotic *A. deanei*, stressing the usability of multivariate analysis in discriminating biomarkers among the lipid classes.

## 2. Results

### 2.1. GC-MS of Derivatizated Fatty Acids

Total fatty acid composition of the *A. deanei* was determined after growth in a variety of conditions (Table S1). Choanomastigotes containing the symbiotic organism were harvested at the exponential growth phase, both without FBS (0% Log) and with 10% FBS (10% Log), as well as at the stationary growth phase without FBS (0% Sta). Aposymbiotic choanomastigotes were also harvested at log phase without FBS (0% Apo) and subjected to lipid extraction and derivatization, as described in the Materials and Methods section. Table 1 shows a predominance of unsaturated fatty acids, namely, C18:3, C18:1, C18:2, C19:1 and C20:3. As shown in Table S1, between the saturated fatty acids, the main component was the unusual branched fatty acid C17:0 (iso-heptadecanoic acid, as confirmed by mass spectrometry and chromatographic analysis) [20,21,22,23], along with C18:0 and another unusual fatty acid, C19 Cyclic (*cis*-9,10- methylene-octadecanoic acid).

The main variations found among the four conditions analyzed were related to MUFA and PUFA levels. As shown in Table 1, the MUFA levels increased for the 10% FBS (10% Log) and aposymbiotic (0% Apo) conditions, whereas the PUFA levels were greater for the 0% FBS Log (0% Log) and 0% FBS collected at the stationary phase (0% Sta). No variations were observed among the four conditions for overall levels of branched-chain fatty acids. This stands in contrast to cyclic fatty acid levels, which increased continuously among the 0% Log, 10% Log, 0% Apo and 0% Sta growth conditions, an increase that occurred with both isomers (*cis*-9,10-methylene-octadecanoic acid and *cis*-11,12-methylene-octadecanoic acid). The condition containing 10% FBS in the growth medium exhibited very similar behavior to that of the 0% Apo.

A comparison of the results of fatty acid analyses for 0% Sta and 0% Log growth conditions reveals changes in the proportion of several individual fatty acids, taking into account that both were grown in the same nutritional conditions. The stationary *A. deanei* culture showed lower proportions of C14, C15Bra, C16, C16:1, C16Bra, C17:1, C18, C20:3, C22, C22:1 and C24 fatty acids, despite the fact that the overall proportion of unsaturated fatty acids remained similar. On the other hand, C17Bra, C18:3, C19Bra and C19Cyc (both isomers) increased in proportion. These results indicate that in the latter stages of growth, the cell must promote the remodeling of lipid synthesis in order to maintain the fluidity of the membrane. Therefore, the fatty acid profile obtained at this phase of growth characterizes the lipidome of this microorganism when the cells are not undergoing division.

### 2.2. NMR Analysis of Lipid Extracts

**Figure 1:**
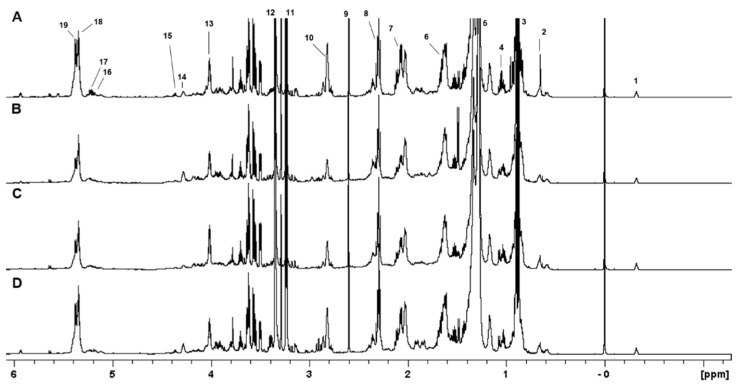
The ^1^H NMR spectra of lipid extracts obtained from *A. deanei* grown in the absence of FBS (**A**), 10% FBS (**B**), aposymbiotic (**C**) and 0% FBS collected at the stationary growth phase (**D**). The chemical shifts are relative to the internal standard TMS (δ = 0.00 ppm). 1−CFA; 2−Erg−C18; 3−R−CH_3_; 4−Erg−C19; 5−(CH_2_)n; 6−Fβ:R−CH_2_−CH_2_−CO; 7−CH=CH−CH_2_−CH−CH (18:1); 8−Fα:R−CH_2_−CH_2_−CO; 9−Suc; 10−CH=CH−CH_2_−CH=CH (18:2); 11−N+(Me_3_)_3_; 12−CHD_2_OD; 13−Gly−C3 (PC/PE); 14−PC−1′; 15−Gly−C1 (DG/TG/PC/PE/PI); 16−Gly−C2 (DG); 17−Gly−C2 (PC/PE/PI); 18−Mono−UFA; 19−Poly−UFA.

**Figure 2:**
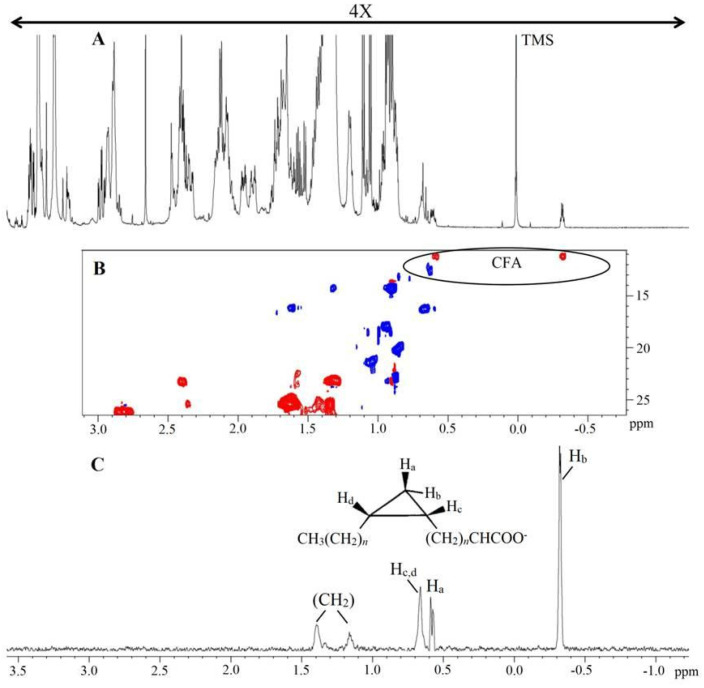
^1^H NMR spectrum of lipid extract obtained from *A. deanei* (**A**), HSQC−ed slice of fingerprint region of CFA (**B**) and 1D selective TOCSY (60 ms) irradiated at H_b_ of CFA (**C**). Chemical shifts are relative to internal standard TMS (δ = 0.00 ppm). The positive phase (blue) corresponds to CH and CH_3_ first-order correlations and the negative phase (red) corresponds to CH_2_ first-order correlations.

**Figure 3:**
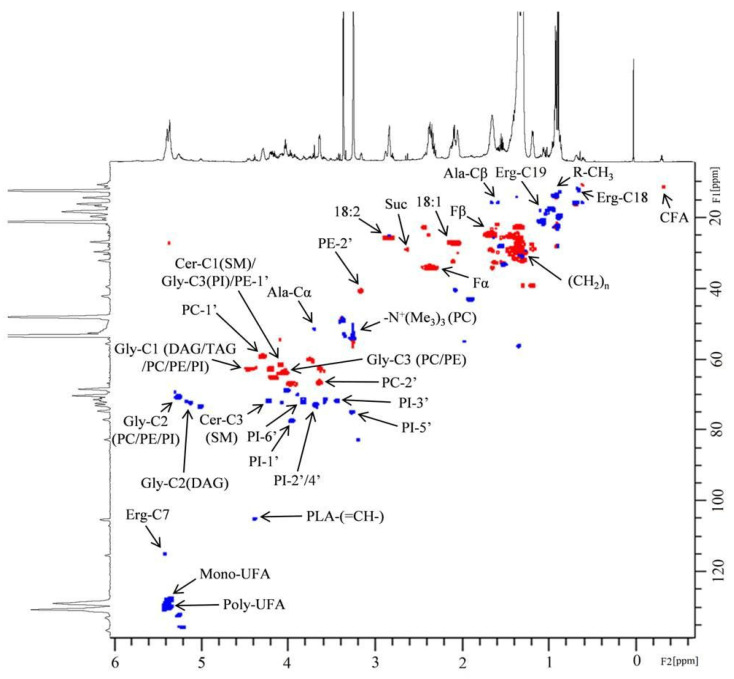
Two-dimensional ^1^H-^13^C multiplicity-edited HSQC NMR correlation map from the lipid extract of *A. deanei* grown in the absence of FBS and the principal lipid assignments. The positive phase (blue) corresponds to CH and CH_3_ first-order correlations and the negative phase (red) corresponds to CH_2_ first-order correlations.

One-dimensional and two-dimensional NMR analysis of the lipid extracts allowed for the identification of the main lipid assignments (Figure 1 and Table S2). As shown in Table 1, the conditions 0% Log and 0% Sta exhibited higher amounts of PUFA, an observation that is confirmed by examining the ^1^H NMR spectra of the four samples (Figure 1). NMR analysis also detected the cyclopropane fatty acid (CFA) at δ −0.32 ppm (Figure 2), which is characteristic of a *cis* CFA ring [24,25,26]. These data were backed by 1D selective TOCSY (Figure 2C), which showed the assignments of CFA ring characteristics of a CFA 19:0 *cis*-9,10-methylene-octadecanoic acid and the *cis*-11,12 isomer [25,26]. Two-dimensional ^1^H-^13^C multiplicity-edited HSQC NMR analyses allowed for the assignment of both ^1^H and ^13^C chemical shift of main lipid classes on the lipid extracts, as shown in Figure 3.

### 2.3. PCA

**Figure 4:**
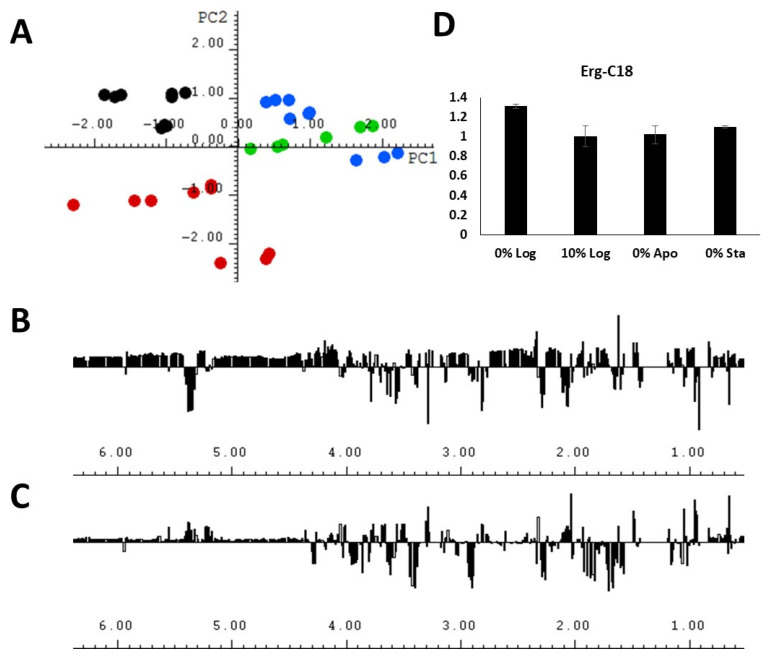
(**A**) The score plots (PC1 vs. PC2) of the PCA analysis performed with the ^1^H NMR spectra of lipid extracts obtained from *A. deanei* grown in the absence of FBS (black dots), 10% FBS (blue dots), aposymbiotic condition (green dots) and 0% FBS collected at the stationary growth phase (red dots); (**B**) 1D loadings plot of PC1; (**C**) 1D loadings plot of PC2; (**D**) relative integral area of the chemical shift of Erg−C18 obtained from the lipid extracts under 4 experimental conditions.

In order to further explore the ^1^H NMR data obtained from the lipid extracts, the authors applied multivariate analysis with the aim of identifying less intense ^1^H NMR assignments that could indicate lipid classes contributing to differentiation. Principal component analysis is a multivariate analysis technique which allows for the correlation between analytical data and experimental conditions [27,28]. PCA was carried out with ^1^H NMR spectra from lipid extracts of *A. deanei* (Figure 4). The four experimental conditions were separated into three distinct groups on the Scores plot (Figure 4A), and both the first (PC1, horizontal axis) and second (PC2, vertical axis) principal components contributed to the differentiation, resulting in a cumulative explained variance of 28.52% and 48.67%, respectively. The 0%Sta experimental condition showed a great PC2 discrimination, and the 1D loadings plot (Figure 4C) indicated the main ^1^H chemical shifts contributing to this separation. The 0%Log condition was separated from the other three conditions mainly by PC1, with chemical shifts assigned to PUFA and MUFA contributing to separation.

An interesting observation was that it was not possible to fully discriminate the conditions 10% Log and 0% Apo by means of PC analysis, whether by PC1 or PC2, or even when a third principal component was plotted (Figure S1). As seen on the Score plot (Figure 4A) for both PC1 and PC2, one of the main chemical shifts that contributed to the differentiation was the signal near 0.65 ppm, which was assigned to Erg−C18, and the integration of this signal (Figure 4D and Table S3) showed no statistically significant difference in the area of this compound for the 10% Log and 0% Apo experimental conditions (Table S4), thus highlighting the usability of multivariate analysis in discriminating biomarkers among the lipid classes.

### 2.4. Sterol Analysis

The relative sterol composition showed a predominance of ergosterol (Supplementary Table S5). *A. deanei* grown in the absence of FBS showed a value of 93.9% for this sterol, whereas *A. deanei* grown in the presence of FBS and aposymbiotic *A. deanei* showed values of 87.0% and 67.5%, respectively (Supplementary Table S5). Low levels of cholesterol were detected—7.8% for *A. deanei* cultivated in the presence of FBS and 11.4% in the aposymbiotic condition. Fecosterol was found only in aposymbiotic *A. deanei* (11.2%).

## 3. Discussion

The current study used NMR and GC-MS techniques to evaluate lipidomic profiles of *A. deanei* with the aim of identifying lipid biochemical changes under different growth conditions. The NMR technique has been used widely for metabolite characterization in the *Trypanosomatidae* family in studies relating to the following: analysis of cell death due to visible mobile lipids in *T. cruzi* epimastigote [29]; metabolic studies of *T. cruzi* [30] and *Crithidia luciliae* [31]; ^13^C NMR analysis of glucose metabolism in *Crithidia fasciculata* [32] and *T. cruzi* [33]; analysis of metabolic end products in *T. cruzi* [34]; the structural characterization of a flagellar calcium-binding protein in *T. brucei* [35]; excreted product analysis using ^1^H NMR of trypanosome isolates [36]; high-resolution ^31^P NMR of *T. brucei*, *T. cruzi* and *Leishmania major* [37] and the characterization of the metabolic responses of mice to *T. brucei brucei* infection using ^1^H NMR [38].

The great similarity between the lipid profiles of *A. deanei* grown with 10% Log and 0% Apo, an observation that was verified by ^1^H NMR and GC-MS analysis, may be explained by the low nutritional requirements of the parasite, which does not even need FBS for growth. Unlike most trypanosomatids, *A. deanei* is not a nutrition-demanding protozoan, requiring only a few amino acids and vitamins supplemented in LIT medium for growth in vitro. Hemin or purine, indispensable for the growth of other trypanosomatids, are unnecessary for *A. deanei* growth [1,9,39]. However, the absence of the endosymbiont influenced the content of lipid components such as ergosterol and fatty acids. McLaughlin [40] did not detect significant changes in fatty acid composition of symbiotic and aposymbiotic cultures of *Crithidia oncopeleti*, although high percentages of unsaturated fatty acids were observed in lipid extracts from *Blastocrithidia culicis* cultures.

A comparison of the 0% Log and 0% Sta cultures (both without FBS) reveals that only the CFA levels increased significantly for the second condition, and the main saturated fatty acids decreased proportionally for the stationary cultures. This result contrasts with that of Bronia [41], who observed an increase in saturated fatty acids and a decrease in mainly polyunsaturated fatty acids in *Trypanosoma cruzi* stationary growth phase cultures.

An analysis of the fatty acid composition of *A. deanei* lipid extracts revealed the main fatty acids to be C18:3, C18:1, iso C17:0, C18:2 and C18:0, which represent nearly 75% of all fatty acids in the four growth conditions analyzed (Table S1). Korn et al. [42] described the fatty acid composition of *Crithidia* sp. (grown in a different culture media) and detected mainly C18:1 (18%), C18:2 (16%) and C18:3 (21%) fatty acids in the lipid extracts. They also found 16% of a (probably) branched-chain fatty acid in *Crithidia sp*., which was not characterized. Similarly, Meyer and Holz [43] showed that the main fatty acids found in some members of the Kinetoplastid order (including *Crithidia* species) were C18:1, C18:2 and C18:3. These authors first reported the presence of CFA C19:0 in *Crithidia* and detected a high percentage (20%) of iso 17:0 fatty acid in the trypanosomatid *Leishmania tarentolae*. 

No studies involving the total fatty acid composition of *A. deanei* were found in the literature. The results presented here are the first to reveal and detect a high iso C17:0 fatty acid content in *Crithidia* (formerly *A. deanei*) genera. As mentioned above, a high C17:0 fatty acid content was detected in *L. tarentolae* and a low C17:0 fatty acid content in epimastigote, trypomastigote and amastigote forms of *T. cruzi* [44]. The authors also identified and quantified other branched-chain fatty acids such as C13, C14, C15, C16 and C19, although smaller amounts of these are present in the lipid extracts. Iso- and anteiso- branched-chain fatty acids are present in the lipid membrane of several bacteria species [45], as well as in some genera, such as *Bacillus* and *Listeria*; they are associated with adaptations involving the maintenance of membrane fluidity at low temperatures or alkaline/acidic pH conditions [46,47]. Although it has been suggested that iso- and anteiso- branched-chain fatty acids play important functions in the development of some *Eukaryote* models [48], the unusually high iso-C17:0 branched-chain fatty acid may represent a strategy used by *A. deanei* to adapt to specific characteristics of insect hosts [49].

In their study involving the presence of CFA C19:0 (*cis*-9,10-methyleneoctadecanoic acid) among many species of the *Trypanosomatidae* family, Fish et al. [25] detected a value of 44% of the CFA in lipid extract from *A. deanei* cultures, thus concluding that this CFA does not seem to be associated with the endosymbiont. Their data were confirmed by the present study, which detected CFA even in aposymbiotic *A. deanei* (Table 1 and Table S1). The presence of the *cis*-11,12 isomer was also observed for the four conditions analyzed.

C19:0 CFA values for the lipid extracts from *A. deanei* obtained during the logarithmic growth phase were considerably smaller than those of the lipid extract obtained from the same organism at the stationary growth phase. Similarly, a high CFA 17:0 percentage was detected in *Pseudomonas putida* at the stationary phase but not at the logarithmic phase [50]. CFAs seem to stabilize membrane lipids against turnover and degradation; in addition, some reports indicate that they contribute to stress tolerance [50,51]. Generally, organisms in which CFAs are found contain greater contents during the later stages of growth [51], thus corroborating the results obtained in the present study. The hypothesis that a correlation exists between high CFA content and stress resistance was tested in *E. coli*., the results showing that CFA synthetase mutants exhibited the same behavior as wild bacteria with regard to stress resistance [25,52]. Therefore, the function of CFA in bacteria and trypanosomatids remains uncertain.

The results for the sterols from *A. deanei* grown in the absence and presence of FBS (Supplementary Table S5) agreed with those published by Korn et al. [53], who observed that *Crithidia fasciculata* contained mainly ergosterol when grown in the absence of FBS, small quantities (6%) of cholesterol when grown in the presence of 20% of FBS and 25% of another sterol (probably 22,23-dihydroergosterol) when grown in the presence of FBS. Palmié-Peixoto et al. [14], who analyzed sterols of *A. deanei* grown with 10% FBS, detected only ergosterol (99%). Fecosterol, methyl-episterol and 4-methyl-ergosta-8,14,24-trienol (detected in this work) were not detected by the authors mentioned above. *A. deanei* is not able to synthesize cholesterol, and a high cholesterol uptake from the culture medium was described for another member of *Trypanosomatidae* family: *T. cruzi*. Epimastigote forms of *T. cruzi* are able to store and mobilize high amounts of cholesterol (52.9%) in reservosome lipid inclusions; however, the cellular implications of lipid uptake are unknown [54]. The present study found that *A. deanei* grown in the presence of FBS exhibits a cholesterol uptake of around 7.8%, a behavior that was slightly affected (increasing to 11.4%) by the removal of the symbiont (Supplementary Table S5). The high ergosterol content observed for aposymbiotic *A. deanei*, together with the high levels of monounsaturated fatty acids, may suggest compensation of the liquid-ordered state of the membrane at physiological temperatures [55].

The authors of this study were able to successfully identify and quantify fatty acids from lipid extracts of *A. deanei* grown in different nutritional and physiological conditions. The GC-MS analysis revealed unusual branched-chain and cyclic fatty acids and provided some potential insights regarding the function of these fatty acids on the physiology of this trypanosomatid. ^1^H NMR analysis confirmed these results, and 2D ^1^H-^13^C HSQC multiplicity-edited NMR analysis provided correlation maps that allowed for the identification of intact lipid classes in the extracts. These NMR fingerprints will greatly aid other research involving lipid analysis in trypanosomatids. Principal component analysis using ^1^H NMR was shown to be a useful tool for identifying the main contributors that distinguish the lipid extracts from various physiological and nutritional conditions. The GC-MS fatty acid profile presented in this work was the first to identify significant amounts of branched-chain fatty acids and cyclic fatty acid isomers in *A. deanei* lipid extracts, and it could point the biochemical synthetic pathways related to these molecules as targets for therapeutic research on protozoan parasites.

## 4. Materials and Methods

### 4.1. Parasite Culture 

The choanomastigote form of *A. deanei* was cultivated in LIT medium [56] supplemented with 10% heat-inactivated fetal bovine serum (FBS) (or, for some experiments, in the absence of FBS) with penicillin and streptomycin, at 28 °C without agitation. Aposymbiotic *A. deanei*, kindly provided by Dr. Stenio Fragoso from the Instituto Carlos Chagas (Curitiba, Paraná, Brazil), was cultured in LIT media in the absence of FBS. Approximately 100 mL of cultures at 1 × 10^8^ choanomastigotes/mL (early stationary phase) and 4 × 10^7^ choanomastigotes/mL (logarithmic phase) were separated by centrifugation at 2000× *g* for 10 min, washed twice with PBS and frozen at −20 °C. All *A. deanei* cultures (except in the log phase experiment) were collected at a density of 1 × 10^8^ choanomastigotes/mL. All cultivation experiments were performed in triplicate.

### 4.2. Lipid Extraction

Parasite pellets were subjected to lysis, being frozen with liquid nitrogen (−196 °C) and returned to a water bath (37 °C) three times, after which they were freeze-dried overnight. A predetermined amount (20 mg for culture) was subsequently transferred to a glass tube with a screw cap and teflon. The parasites were then extracted using a (1:1 *v*/*v*) chloroform–methanol mixture (both obtained from Tedia, Fairfield, OH, USA) at 100 °C for 2 h [57,58], after which the lipid extracts were centrifuged at 8000× *g* for 15 min, the organic solvent in the supernatant was collected and the analyses were performed.

### 4.3. Nuclear Magnetic Resonance (NMR)

After drying by a N_2_ stream, the samples were deuterium-exchanged by repeated dissolution in MeOD-D_2_O (2:1) and freeze-dried. Their spectra were obtained from solutions in MeOD-CDCl_3_ (1:1) at 30 °C, using tetramethylsilane (TMS) as reference (δ = 0). All spectra were obtained using either a Bruker 400 MHz Avance III NMR spectrometer with a 5 mm BBI inverse probe with a Z gradient or a Bruker 600 MHz Ascend NMR spectrometer with a 5 mm QXI inverse probe with a Z gradient. One-dimensional ^1^H-NMR was performed using 64 scans to give a Signal/Noise (S/N) ratio of at least 2000/1 (90° pulse, relaxation delay = 4.0 s, number of time domain points = 65,536 and acquisition time = 7.7 s); experiments were performed without tube rotation, with the TMS signal at a medium width varying from 0.8 to 1.0 Hz, the ^1^H measurements being obtained in triplicate. Two-dimensional NMR experiments were carried out using edited-HSQC, heteronuclear correlation via double inept transfer with decoupling during acquisition, using trim pulses in inept transfer with multiplicity editing during the selection step (hsqcedetgpsisp2.2), TOCSY, total homonuclear correlation via Hartman-Hahn transfer using the MLEV17 sequence for mixing, using a mixing time of 0.06 s (mlevphpr.2) and HMBC heteronuclear correlation via zero and double quantum coherence, optimized on long-range couplings’ (hmbcgplpndqf) pulse sequences. The 2D experiments were recorded for quadrature detection in the indirect dimension, and edited-HSQC spectra were acquired using 128 scans per series of 1 K × 256 W data points with zero filling in F1 (4 K) prior to Fourier transformation [28,59]. Data processing and integration were performed using the software TOPSPIN 3.1 (Bruker Biospin, Rheinstetten, Germany).

### 4.4. Principal Component Analysis (PCA) and Data Reduction 

After the NMR spectral phase and baseline correction, each spectrum was data-reduced to 440 regions of equal width (0.01 ppm) using the AMIX (Analysis of MIXtures) software package, version 3.8 (Bruker Biospin, Rheinstetten, Germany). The spectral width considered was from 0.5 to 6.50 ppm. The spectral region close to the CHD_2_OD resonances (δ 3.32–3.36) was removed from all data sets prior to normalization and multivariate data analysis in order to eliminate variation due to water suppression efficiency or solvent multiplicity in homogeneities. Following a preliminary PCA, the regions corresponding to R-CH_3_ (δ 0.84–0.91), (CH_2_)_n_ (δ 1.2–1.4), Ala-Cβ (δ 1.50–1.57), succinate (δ 2.58–2.61) and N+(CH_3_)_3_ (δ 3.19–3.25) resonances were also removed from some of the spectra, mainly because minor peak shape deformations in this region interfere with PCA due to the high intensity of the signals in this region. All remaining frequency regions of the spectra were analyzed with Pareto scaling.

### 4.5. Lipid Derivatizations 

Aliquots of 100 µL of samples dissolved in CHCl_3_-MeOH (1:1, *v*/*v*) were first dried under a gentle N_2_ stream, after which methanolysis was carried out with 1N methanolic HCl (Sigma-Aldrich, St. Louis., MO, USA) at 100 °C for 2 h to obtain the fatty acid methyl esters (FAMEs). The resulting FAMEs were extracted by a partition between n-hexane (1 mL) and distilled water (0.5 mL). The organic (upper) phase was collected and evaporated under a stream of N_2_. In order to analyze the sterols, 50 µL of the lipid extract was dried under a gentle N_2_ stream and derivatized for 30 min in a sealed vial with N,N-bis(trimethylsilyl)-2,2,2-trifluoroacetamide (BSTFA)-pyridine (2:1, *v*/*v*) (Sigma-Aldrich) at 100 °C. The trimethylsilyl derivatives were then subjected to GC-MS analysis.

### 4.6. Gas Chromatography Coupled with Mass Spectrometry (GC-MS) 

GC-MS analysis was carried out using a Varian 3800 gas chromatograph coupled to a 4000 MS detector (ion trap), equipped with a 30 m × 0.25 mm i.d. low-bleed/MS capillary column (VF-5ms) (both from Agilent/Varian, Santa Clara, CA, USA). The temperature ramp to fatty acid analysis was as follows: injector 250 °C, oven initially at 100 °C, maintained for 2 min, heated to 280 °C (5 °C/min) and then maintained for 2 min. The temperature ramp was altered as follows for sterol analysis: injector 250 °C, column maintained at 200 °C for 1 min, increased to 300 °C at a rate of 15 °C/min and finally maintained at 300 °C for 15 min [60]. For all analyses, the injection volume was 1 µL, with a split ratio of 1:10. Post-run analysis was performed using a Saturn Workstation 5.1 [61]. GC-MS experiments were performed in triplicate.

### 4.7. Cell Microscopy

Coanomastigotes grown under different conditions were fixed with methanol at room temperature for 15 min and stained with Giemsa. The slides were then mounted and analyzed under an Olympus microscope equipped with a 100× Oil objective.

### 4.8. Data Analysis

Raw data values obtained from ^1^H NMR integration and GC-MS chromatograms were analyzed using the data analysis tool from Microsoft Excel 2019 software. The TMS signal was normalized to the value of 1.00 on all ^1^H NMR spectra, and the relative fatty acid proportion was calculated by means of the integration and sum of the peaks corresponding to all fatty acids on each GC-MS chromatogram, normalized to 100%. Unless otherwise stated, the data in the tables are shown as mean minus/plus standard deviation. The statistical significance of the differences among growth conditions was calculated using one-way ANOVA. The *p*-values obtained were adjusted to account for multiple comparisons.

## Figures and Tables

**Table 1:** The relative proportion of the fatty acid class of total lipid extracts from *A. deanei* under the four growth conditions, namely, 0% FBS collected at the exponential growth phase (0% Log); 10% FBS collected at the exponential growth phase (10% Log); 0% FBS aposymbiotic (0% Apo) and 0% FBS collected at the stationary growth phase (0% Sta).

Fatty Acid Class	0% Log	10% Log	0% Apo	0% Sta
SFA	31.67	31.54	31.86	30.12
UFA	68.33	68.46	68.14	69.88
MUFA	31.07	35.16	34.40	30.90
PUFA	28.69	25.65	25.64	30.26
BFA	11.30	12.82	11.69	12.39
CFA	6.53	7.07	7.72	8.67
UFA/SFA	2.16	2.17	2.14	2.32

SFA: saturated fatty acids; UFA: unsaturated fatty acids; MUFA: monounsaturated; PUFA: polyunsaturated; BFA: branched-chain fatty acids; CFA: cyclic fatty acids.

## Data Availability

Data will be made available upon request due to privacy.

## Supplementary Materials

The following supporting information may be downloaded at https://www.mdpi.com/article/10.3390/molecules29143352/s1: Table S1: Changes in fatty acid methyl ester ratios obtained from lipid extracts from *A. deanei* under the four growth conditions; Table S2: ^1^H NMR and ^13^C NMR lipid assignments performed on the lipid extracts of *A. deanei*; Table S3: ^1^H NMR lipid assignments and integration values from lipid extracts from *A. deanei* under the four growth conditions; Figure S1: Scores plot of PCA performed using ^1^H NMR spectra from lipid extracts of *A. deanei* under four experimental conditions; Table S4. Statistical significance of relative levels of Ergosterol obtained through integration of ^1^H NMR chemical shift corresponding to lipid assignment on each growth condition; Table S5: The sterol composition percentages of *A. deanei* grown in the presence and absence of FBS and in the aposymbiotic condition.
