# Supplementary material for: Lipidomic Analysis Reveals Branched-Chain and Cyclic Fatty Acids from Angomonas deanei Grown under Different Nutritional and Physiological Conditions
Source: Molecules. 2024 Jul 17;29(14):3352. doi: 10.3390/molecules29143352 (PMC11280109; doi:10.3390/molecules29143352)
Supplement: Supplementary file 1 [file molecules-29-03352-s001.zip › SUPPLEMENTARY MATERIAL.pdf]

SUPPLEMENTARY MATERIAL

Lipidomic analysis reveals branched-chain and cyclic fatty acids from *Angomonas deanei* grown under different nutritional and physiological conditions.

Arquimedes Paixão Santana-Filho, Aramís José Pereira, Letícia Adejani Laibida, Normanda Souza-Melo, Wanderson Duarte DaRocha * and Guilherme Lanzi Sassaki *

Departamento de Bioquímica e Biologia Molecular, Universidade Federal do Paraná, Curitiba 81531-980, PR, Brazil; santana@ufpr.br (A.P.S.-F.); aramis.jpereira@yahoo.com.br (A.J.P.); leticia_laibida@yahoo.com.br (L.A.L.); normandasouzamelo@gmail.com (N.S.-M.)

* Correspondence: darocha@ufpr.br (W.D.D.); sassaki@ufpr.br (G.L.S.)

Table S1. Changes in fatty acid methyl esters ratios obtained from lipid extracts from *A. deanei* under the four growth conditions: 0% FBS collected at the exponential growth phase (0% Log); 10% FBS collected at the exponential growth phase (10% Log); 0% FBS collected at the stationary growth phase (0% Sta) and 0% FBS aposymbiotic (0% Apo)

Fatty Acid	0% Log		10% Log		0% Apo		0% Sta	
C12	0.03 ±	0.0043	0.02 ±	0.0018	0.04 ±	0.0054	0.02 ±	0.0014
C13Bra	0.04 ±	0.0083	0.03 ±	0.0007	0.03 ±	0.0078	0.02 ±	0.0031
C14	1.18 ±	0.0357	0.82 ±	0.0105	1.32 ±	0.0847	0.85 ±	0.0207
C14Bra	0.01 ±	0.0018	0.01 ±	0.0003	0.01 ±	0.0029	0.00 ±	0.0009
C15	0.05 ±	0.0059	0.06 ±	0.0063	0.06 ±	0.0022	0.05 ±	0.0104
C15Bra	0.41 ±	0.0538	0.26 ±	0.0078	0.29 ±	0.0629	0.30 ±	0.0751
C16	2.71 ±	0.0581	2.45 ±	0.0624	2.29 ±	0.1330	2.12 ±	0.2620
C16:1	3.29 ±	0.2396	3.31 ±	0.0646	2.24 ±	0.0802	2.26 ±	0.4086
C16Bra	0.22 ±	0.0384	0.44 ±	0.0158	0.60 ±	0.0000	0.18 ±	0.0047
C17	0.16 ±	0.0060	0.25 ±	0.0253	0.95 ±	0.1181	0.15 ±	0.0239
C17:1	0.73 ±	0.0069	0.85 ±	0.0519	1.03 ±	0.0359	0.53 ±	0.1005
C17Bra	9.00 ±	0.6961	10.02 ±	1.1719	9.75 ±	1.1801	9.95 ±	0.8765
C18	8.55 ±	0.2085	6.30 ±	0.3941	6.32 ±	0.9240	4.81 ±	0.5492
C18:1	19.00 ±	0.2838	21.60 ±	1.0068	20.85 ±	1.4144	20.69 ±	0.3261
C18:2	8.57 ±	0.4428	7.65 ±	0.2775	8.10 ±	1.5201	8.73 ±	0.8926
C18:3	21.35 ±	0.7220	21.91 ±	1.1273	20.71 ±	1.8270	25.00 ±	1.2718
C19	0.30 ±	0.0146	0.53 ±	0.0066	0.56 ±	0.2890	0.30 ±	0.0811
C19:1	6.15 ±	0.1483	6.46 ±	0.3484	7.30 ±	1.6135	6.09 ±	0.2428
C19Bra	1.63 ±	0.2114	2.06 ±	0.0260	1.00 ±	0.5072	1.94 ±	0.1675
C19Cyc-9,10	4.84 ±	0.1225	5.16 ±	0.1744	5.71 ±	0.7394	6.25 ±	0.5533
C19Cyc-11,12	1.69 ±	0.1070	1.91 ±	0.0743	2.01 ±	0.4212	2.42 ±	0.4607
C20	0.20 ±	0.0147	0.28 ±	0.0238	0.23 ±	0.0492	0.21 ±	0.0401
C20:1	0.21 ±	0.0038	0.52 ±	0.0109	0.32 ±	0.0821	0.18 ±	0.0072

C20:2	0.23 ±	0.0144	0.40 ±	0.0079	0.36 ±	0.0485	0.23 ±	0.0425
C20:3	7.12 ±	0.7558	3.34 ±	0.6917	4.57 ±	0.0000	5.02 ±	1.9577
C22	0.21 ±	0.0831	0.49 ±	0.0652	0.27 ±	0.0028	0.11 ±	0.0242
C22:1	1.69 ±	0.1152	2.41 ±	0.0873	2.66 ±	0.2087	1.15 ±	0.0824
C23	0.02 ±	0.0003	0.06 ±	0.0297	0.04 ±	0.0053	0.13 ±	0.0351
C24	0.44 ±	0.1324	0.40 ±	0.0578	0.37 ±	0.0113	0.34 ±	0.0105

Table S2. ^{1}H NMR and ^{13}C NMR lipid assignments performed on the lipid extracts of *A. deanei*. Abbreviations: Ala, alanine; Cer, ceramide; CFA, cyclopropane fatty acid; DAG, diacylglycerol; Erg, ergosterol; F*α*, fatty acid alfa carbon; Fβ, fatty acid beta carbon; Gly, glycerol; PC, phosphatidylcholine; PE, phosphatidylethanolamine; PI, phosphatidylinositol; PLA, plasminogen; SM, sphingomyelin; Suc, succinate; TAG, triacylglycerol; UFA, unsaturated fatty acids.

Lipid class/compound	δ (ppm) ^{1}H /^{13}C
CFA	-0.32/11.00
Erg-C18	0.65/12.01
R-CH_3	0.84-0.91/14.08
Erg-C19	1.06/18.71
$(CH_2)_n$	1.20-1.41/29.62
Ala-Cβ	1.48-1.54/16.81
Fβ:R-*CH_2*-CH_2-COOH	1.56-1.67/25.2
-CH=CH-*CH_2*-CH-CH-(18:1)	2.01-2.03/27.44
F*α*: R-CH_2-*CH_2*-COOH	2.27-2.39/34.37
Suc	2.60/29.30
-CH=CH-*CH_2*-CH=CH-(18:2)	2.76-2.86/25.93
PE-2′	3.14/40.88
-$N^+(Me_3)_3$ (PC)	3.19-3.25/54.25
PI-5′	3.24/75.13
CHD_2OD	3.34/49.01
PI-3′	3.42-3.43/71.85
Ala-C*α*	3.56-3.60/50.92
PC-2′	3.63/66.82
PI-4′	3.64/73.01
PI-2′	3.66/73.03
PI-6′	3.80-3.83/72.37
PI-1′	3.88/77.26
Gly-C3(PC/PE)	4.01/61.84
Cer-C1(SM)/Gly-C3(PI)/PE-1′	4.03-4.04/61.87
Cer-C3(SM)	4.18/72.09
PC-1′	4.27/59.46
PLA-(=CH-)	4.36/108.07
Gly-C1(DAG/TAG/PC/PE/PI)	4.18;4.44/63.02
HOD/CD_3OD	4.57
Gly-C2 (DAG)	5.17/72.13
Gly-C2 (PC/PE/PI)	5.25/70.88
Mono-UFA	5.34/128.36
Poly-UFA	5.36/130.28
Erg-C7	5.38/116.72

Table S3. ^{1}H NMR lipid assignments and integration values from lipid extracts from *A. deanei* under the four growth conditions: 0% FBS collected at the exponential growth phase (0% FBS); 10% FBS collected at the exponential growth phase (10% FBS); 0% FBS collected at the stationary growth phase (0% Sta) and 0% FBS aposymbiotic (0% Apo). Results of the four experimental conditions were normalized by the TMS area.

δ (ppm)	Lipid assignments	0% Log	10% Log	0% Apo	0% Sta
**0.00**	TMS	1	1	1	1
**0.6252**	Erg-C18	1.308 ± 0.024	1.002 ± 0,109	1.020 ± 0,091	1.097 ± 0,009
**1.641**	Fβ-R**CH2**CH2CO	5.990 ± 0,007	5.619 ± 0,148	5.327 ± 0,066	7.309 ± 0,006
**2.06**	**CH2**CO(20:4)/ CH=CHCH2CH=CH(18:1;18:2;20:4)	6.683 ± 0,006	4.655 ± 0,100	4.549 ± 0,060	6.645 ± 0,004
**2.352**	Fα-RCH2**CH2**CO/ CH=CH**CH2CH2**CO (22:6)	5.008 ± 0,006	4.571 ± 0,073	4.357 ± 0,069	5.549 ± 0,005
**2.853**	-CH=CH-(**CH2**-CH=CH)y (20:4; 22:6)/ -CH=CH-**CH2**-CH=CH- (18:2)	3.595 ± 0,009	2.016 ± 0,048	1.939 ± 0,088	3.758 ± 0,009

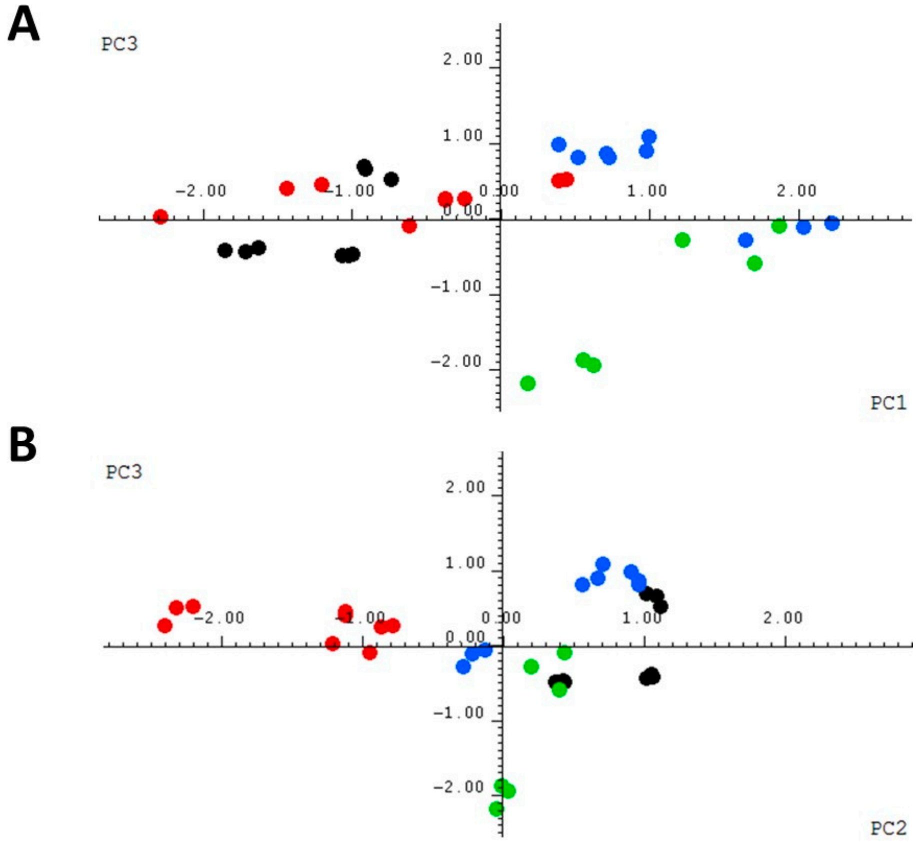

Figure S1: Scores plot of PCA performed using 1H NMR spectra from lipid extracts of A. deanei under the four experimental conditions. (A): PC1 versus PC3; (B) PC2 versus PC3.

Table S4. Statistical significance of measured levels of Ergosterol obtained through integration of ^{1}H NMR chemical shift corresponding to this lipid assignment on each growth condition.

	10% Log	0% Apo	0% Sta
0% Log			
10% Log			
0% Apo			

p≤ 0.001
p≤0.01
n.s.

Table S5. Sterols percentual composition of *A. deanei* grown in the presence and absence of FBS and aposymbiotic.

Sterols	*A.deanei*-10%[a]	*A.deanei*[b]	*A.deanei*-A[c]
cholesterol	7.8	-	11.4
ergosterol	87.0	93.9	67.5
fecosterol	-	-	11.2
4-methyl-ergosta-8,14,24-trienol	3.2	6.0	9.9
methyl-episterol	1.90	-	-

[a]- *A. deanei* grown in the presence of FBS 10%.
[b]- *A. deanei* grown in the absence of FBS.
[c]- Aposymbiotic *A. deanei*.
